# Supplementary material for: Intrastromal graft of anterior lens capsule. A substitute for Bowman layer graft transplantation for keratoconus
Source: PLoS One. 2024 Nov 19;19(11):e0306015. doi: 10.1371/journal.pone.0306015 (PMC11575790; doi:10.1371/journal.pone.0306015)
Supplement: S1 Table — (PDF) [file pone.0306015.s001.pdf]

S1 Table. Changes in central corneal thickness (CCT) following the transplantation of an anterior lens capsule (ALC) graft during the follow-up period.

CCT	Time	Mean	Standard deviation	Minimum	Maximum
**Apex**	0	340	26.4	313	376
	7	342	26.6	325	381
	14	354	32.5	320	398
	21	354	24.2	322	380
	28	357	27.1	326	392
**0-2 mm**	0	340	27.7	312	378
	7	342	27.0	324	382
	14	355	31.8	320	395
	21	353	23.6	324	380
	28	358	28.5	327	396
**2-5 mm**	0	336	26.0	310	372
	7	339	30.7	321	385
	14	339	24.4	319	370
	21	353	26.4	321	385
	28	356	25.6	324	386
