# Supplementary material for: Intrastromal graft of anterior lens capsule. A substitute for Bowman layer graft transplantation for keratoconus
Source: PLoS One. 2024 Nov 19;19(11):e0306015. doi: 10.1371/journal.pone.0306015 (PMC11575790; doi:10.1371/journal.pone.0306015)
Supplement: S2 Table — (PDF) [file pone.0306015.s002.pdf]

S2 Table. Changes in K-readings following the transplantation of an anterior lens capsule (ALC) graft during the follow-up period.

Curvature	Time	Mean	Standard deviation	Minimum	Maximum
**K1**	0	48.8	2.2	46.4	51.7
	7	46.9	2.9	42.7	49.2
	14	46.1	4.34	41.1	51.6
	21	48.9	2.46	45.7	51.6
	28	45.9	4.27	41.3	50.6
**K2**	0	50.6	0.85	50.2	51.9
	7	51.4	3.30	48.8	55.8
	14	48.7	2.59	45.9	52
	21	50.3	1.26	48.8	51.8
	28	49.1	1.43	47.7	51
**Km**	0	49.6	1.53	48.2	51.8
	7	49.0	2.79	45.6	52.3
	14	47.3	3.54	43.3	51.8
	21	49.6	1.84	47.2	51.7
	28	47.4	2.89	44.6	50.8
