# Supplementary material for: Intrastromal graft of anterior lens capsule. A substitute for Bowman layer graft transplantation for keratoconus
Source: PLoS One. 2024 Nov 19;19(11):e0306015. doi: 10.1371/journal.pone.0306015 (PMC11575790; doi:10.1371/journal.pone.0306015)
Supplement: Attachment — Submitted filename: Response to Reviewers.docx [file pone.0306015.s003.docx]

Carlos A Rodríguez Barrientos

Escuela Superior de Oftalmología. Instituto Barraquer de América

Bogotá, Colombia.

**PLOS ONE**

Rebuttal letter of reviewer’s comments on “Intrastromal graft of anterior lens capsule. A substitute for Bowman layer graft transplantation for keratoconus”. PONE-D-24-23473.

**Dear Editor:**

We would like to express our gratitude to the reviewers for their valuable comments and suggestions on our manuscript titled “Intrastromal graft of anterior lens capsule. A substitute for Bowman layer graft transplantation for keratoconus”. We sincerely appreciate the time and effort they dedicated to reviewing our work. In response to their insightful feedback, we have made substantial revisions to enhance the quality and scientific rigor of our study.

**Reviewer 1:** We are grateful to Reviewer 1 for the positive comments to our study.

**Reviewer 2:** We are grateful to Reviewer 2 for their very constructive feedback. In their comment regarding be clear in the abstract and in the methodology that the ALC is taken from porcine eye. We have taken the suggestion into account and included in the abstract (line 22 and 27), and in the Material and methods section, line 122 mentions “Four fresh porcine eyes…”. In this section in line 127, we mention that grafts were procured within 24 hours after porcine donor death.

Also, Reviewer 2 highlighted the need to mention the possibility of taking ALC graft from cataract patients undergoing femto cataract surgery. The recommendation has been beneficial and interesting. We have taken this suggestion into account and included in the Discussion section, lines 379 to 388. Finally, the “donor size” topic is another interesting observation mentioned by Reviewer 2, in response to this suggestion, we include this idea in the Discussion section, line 390 to 395, and in a search of this topic it is known to us that no information is reported. We believe this addition strengthens the overall scientific rigor of our work.

Overall, we would like to emphasize our commitment to ensuring the scientific integrity and validity of our research. We have carefully considered each of the reviewers' suggestions and have made the necessary revisions to address their concerns.

We kindly request that you reconsider our revised manuscript for publication in PLOS ONE. We express our gratitude to the reviewers once again for their valuable input, which has undoubtedly enhanced the overall quality of our work.

Thank you for your consideration.

Sincerely,

Carlos A Rodríguez-Barrientos. MD, PhD

Escuela Superior de Oftalmología del Instituto Barraquer de América. Bogotá, Colombia.
